# Development of Functional Nanomaterials for Applications in Chemical Engineering

**DOI:** 10.3390/nano13030609

**Published:** 2023-02-03

**Authors:** Meiwen Cao

**Affiliations:** State Key Laboratory of Heavy Oil Processing, Department of Biological and Energy Chemical Engineering, College of Chemistry and Chemical Engineering, China University of Petroleum (East China), Qingdao 266580, China; mwcao@upc.edu.cn

Nanomaterials are materials with particle sizes of less than 100 nm in at least one of their dimensions. The special structure of nanomaterials endows them unique characteristics different from those of bulk materials and individual atoms, which are known as surface effect, quantum size effect, and macroscopic quantum tunneling effect, etc. Therefore, nanomaterials usually exhibit novel optical, acoustic, electrical, magnetic, thermal, and catalytic properties, and have been widely applied in various fields. Nowadays, the production and application of nanomaterials in the chemical industry are very common, especially in the fields of new catalysts, selective adsorbents, corrosion prevention coatings, environmental protection, and other biopharmaceuticals and medical instruments, playing important roles in promoting the development of society and human beings.

This Special Issue, Nanomaterials for Chemical Engineering, focuses on the development of functional nanomaterials in the chemical engineering field. It collects 14 original research papers and 2 comprehensive review papers by the excellent scientists from relevant fields, covering the topics of development of novel nanomaterials and synthesis methods, experimental characterization, and computational modeling studies, as well as exploitation in devices and practical applications.

The synthesis, characterization, and property investigation of new nanomaterials are eternal prerequisites to meet the needs of nanotechnology development for chemical engineering applications. Sazanova et al. [1] reported the synthesis and characterization of zinc oxide (ZnO) nanoparticles by plasma-enhanced chemical vapor deposition, establishing that the synthesizing parameters of zinc source temperature and reactor temperature can effectively control the size and morphology of ZnO nanostructures. Gaur and coworkers [2] synthesized SnS_2_ nanoparticles using a thermal decomposition approach and produced novel morphologies (e.g., nanoparticles, nanoplates, and flower-like morphologies assembled from flakes) by using different alkylamines as capping agents. Shao and coworkers [3] synthesized the Ag@MXene hybrids and studied their nonlinear optical characteristics, making great contributions to the development of ultrathin optoelectronic nanodevices and optical limiters. Liu et al. [4] fabricated 2D R-P type (PEA)_2_(MA)_2_Pb_3_I_10_ perovskite films on quartz substrates and studied their terahertz and ultrafast photoelectric response characteristics, demonstrating their potential applications in solar cells and photoelectric devices. Wang and coworkers [5] prepared methyl-modified ZrO_2_-SiO_2_ (ZrO_2_-MSiO_2_) membranes via the sol–gel method and characterized their physical–chemical properties. With excellent hydrothermal stability and regeneration capability, the ZrO_2_-MSiO_2_ membranes have significant potential in steam-stable hydrogen permselective applications. By incorporating Gyrotactic microbes to prevent the bioconvection of small particles and to improve consistency, Ali et al. [6] discussed the relevance of Lorentz and Coriolis forces on the kinetics of gyratory Maxwell nanofluids flowing against a continually stretched surface, contributing to the areas of elastomers, mineral productivity, paper-making, biosensors, and biofuels.

Many nanomaterials are highly porous in structure and have high specific surface area, which can be used for gas adsorption in the aims of purification and/or storage. Two research works in this Special Issue studied the adsorption and storage of natural gases on defined nanomaterials. [7,8] First, Shkolin et al. [7] synthesized an active carbon nanomaterial from cheap peat raw materials and investigated its structural–energetic and adsorption properties towards natural gas (ANG). The study provides insights into the solution for improving the safety and storage capacity of low-pressure gas storage systems. Second, Zhang et al. [8] reported the CH_4_ adsorption properties on extended line defect (ELD) graphene according to the first principles of density functional theory (DFT). The Mn modification of ELD graphene was found to significantly affect the CH_4_ adsorption. The specific molecular configurations and adsorption behaviors were discussed in detail in the paper. In another paper, Sodha et al. [9] presented a comprehensive review on zeolite-based nanocomposites for the treatment of effluents from wastewater. The review provides the basic knowledge about zeolites and highlights the types, synthesis, and removal mechanisms of zeolite-based materials for wastewater treatment along with the research gaps, being helpful for worldwide research on this topic.

Nanomaterials as applied as catalysts play important roles in many chemical engineering fields, being able to control reaction time, improve reaction speed and efficiency, save resources and energy, and improve economic benefits. Therefore, the study on catalytic nanomaterials attracts great research interests. The Special Issue presents two works focusing on fabrication of CeO_2_-composited catalysts for maleic anhydride hydrogenation (MAH). Zhang et al. [10] prepared CeO_2_-supported Ni catalysts with different Ni loadings and particle sizes by the impregnation method and investigated their hydrogenation performance. The work provides a theoretical and experimental basis for the preparation of high-activity catalysts for MAH. Liu et al. [11] synthesized CeO_2_ supports with various shapes (e.g., nanocubes, nanorods, and nanoparticles) by using the hydrothermal technique, which were employed for supporting Ni species as catalysts for MAH. The study demonstrated morphology-dependent performances of the Ni/CeO_2_ catalysts, which is helpful for developing novel catalysts for MAH. In another work, Li and coworkers [12] reported the synthesis of a series of Mg–Zr composite oxide catalysts by the hydrothermal method, aiming to catalyze the transesterification of glycerol with dimethyl carbonate to produce glycerol carbonate. The effects of the preparation method and Mg/Zr ratio on the catalytic performance and the deactivation of the catalysts were systematically investigated and discussed.

Nanomaterials have also found extensive applications in the fields of daily chemical products, cosmetics, pesticides, biomedicine etc., where the nanomaterials can be used as nanocarriers to load various active components for targeted delivery and controlled release. Rocha et al. [13] performed a systematic study on polymeric particles based on gelatin and poly-*ε*-caprolactone (PCL) containing essential oil from *Lippia origanoides*. The developed biocides have high physical stability and particle surface microtexture as well as pronounced bioactivity, which are efficient alternative controlling agents of *Conotrachelus humeropictus* and *Moniliophtora perniciosa*, the main pests of *Theobroma grandiflorum*. Yang et al. [14] presented a comprehensive review on peptide-based nanocarriers for gene delivery. The review puts forward discussion on the biological barriers for gene delivery, the peptide molecular design and assembly with DNA, the targeted delivery and controlled release of genome, the structure–function relationships of the delivery systems, and the current challenges and future perspectives in related fields, providing guidance towards the rational design and development of nonviral gene delivery systems.

Nanomaterials can also be engineered into the industrial production so as to improve the production process, elevate production efficiency, and optimize product performance. The work by Ali and coworkers [15] studied the effect of inlet flow strategies on the dynamics of pulsed fluidized bed of nanopowder. They changed the conventional single-drainage (SD) flow strategy to the modified double-drainage (MDD) flow strategy, which improved the production process by purging the primary flow during the non-flow period of the pulse to eliminate pressure buildup in the inlet flow line while providing a second drainage path to the residual gas. Sfameni et al. [16] reported the development of an efficient and eco-friendly procedure to form highly hydrophobic surfaces on cotton fabrics. By using a two-step treatment procedure, that is, first producing a hybrid silane film on cotton fabrics and then modifying with low-surface-energy components, the cotton fabrics were endowed with excellent water repellency. The work provides a new sustainable approach for fabric finishing and treatment.

In short, this Special Issue is expected to be interesting and enriching for readers by virtue of featuring all of the abovementioned high-quality original research works and comprehensive review papers. We give our sincere thanks to the excellent scholars that have made contributions. Currently, we are developing the second volume of the Special Issue, that is, “Nanomaterials for Chemical Engineering II”. We welcome more excellent scholars to submit their excellent works in the area of synthesis and application of functional nanomaterials for chemical engineering.
